# PINK1 and Parkin mitochondrial quality control: a source of regional vulnerability in Parkinson’s disease

**DOI:** 10.1186/s13024-020-00367-7

**Published:** 2020-03-13

**Authors:** Preston Ge, Valina L. Dawson, Ted M. Dawson

**Affiliations:** 1 Neuroregeneration and Stem Cell Programs, Institute for Cell Engineering, Department of Neurology, Department of Physiology, Solomon H. Snyder Department of Neuroscience, Department of Pharmacology and Molecular Sciences, Johns Hopkins University School of Medicine, 733 North Broadway, Suite 731, Baltimore, MD 21205 USA; 2 Adrienne Helis Malvin Medical Research Foundation, New Orleans, LA 70130 USA; 3 Diana Helis Henry Medical Research Foundation, New Orleans, LA 70130 USA; 4 Present address: Department of Brain and Cognitive Sciences, Massachusetts Institute of Technology, Cambridge, MA 02139 USA; 5 Present address: Picower Institute for Learning and Memory, Cambridge, MA 02139 USA; 6 Present address: Harvard-MIT MD/PhD Program, Harvard Medical School, Boston, MA 02115 USA

**Keywords:** Parkinson disease, Parkin, PINK1, Mitochondria, Mitophagy, Selective vulnerability, Substantia nigra

## Abstract

That certain cell types in the central nervous system are more likely to undergo neurodegeneration in Parkinson’s disease is a widely appreciated but poorly understood phenomenon. Many vulnerable subpopulations, including dopamine neurons in the substantia nigra pars compacta, have a shared phenotype of large, widely distributed axonal networks, dense synaptic connections, and high basal levels of neural activity. These features come at substantial bioenergetic cost, suggesting that these neurons experience a high degree of mitochondrial stress. In such a context, mechanisms of mitochondrial quality control play an especially important role in maintaining neuronal survival. In this review, we focus on understanding the unique challenges faced by the mitochondria in neurons vulnerable to neurodegeneration in Parkinson’s and summarize evidence that mitochondrial dysfunction contributes to disease pathogenesis and to cell death in these subpopulations. We then review mechanisms of mitochondrial quality control mediated by activation of PINK1 and Parkin, two genes that carry mutations associated with autosomal recessive Parkinson’s disease. We conclude by pinpointing critical gaps in our knowledge of PINK1 and Parkin function, and propose that understanding the connection between the mechanisms of sporadic Parkinson’s and defects in mitochondrial quality control will lead us to greater insights into the question of selective vulnerability.

## Background

Parkinson’s disease (PD) is a late-onset neurodegenerative disease characterized by a core triad of symptoms; resting tremor, bradykinesia, and elevated resting tone [1]. While 10% of patients carry single gene mutations that cause PD (monogenic PD), over 90% of patients have no known family history or known genetic cause of their disease (sporadic PD, or sPD) [1]. PD has traditionally been viewed as a disease caused by the selective degeneration of dopamine (DA) neurons found in the substantia nigra pars compacta (SNpc) due to early findings that SNpc degeneration is the most consistent postmortem finding in patient brains, that dopamine replacement through L-DOPA is an effective management strategy for the motor symptoms, and that the selective SNpc DA neuron toxin MPTP recapitulates the clinical phenotype of PD [2]. However, systematic, large-scale characterization of postmortem PD brains has provided a contrasting image of disease progression based on the presence of Lewy bodies (LB), large aggregates of misfolded α-synuclein protein, which has served as a canonical marker of disease pathology for decades [2, 3]. Pathologic staging of α-synuclein-positive LBs has revealed widespread involvement of most major subdivisions of the central nervous system (CNS), ranging from brainstem nuclei to cortex [3, 4]. A growing body of evidence in both human patients and preclinical animal models suggests that LBs may initially appear in the brainstem or enteric nervous system and spread across the brain in a prion-like manner [5–12].

The realization that PD pathology is not solely confined to the SNpc and can spread across the CNS has profoundly altered our understanding of PD pathogenesis and disease progression. However, despite evidence of widespread pathology, not all cell populations are equally resilient. Certain populations of neurons remain more vulnerable to developing LB pathology and to neurodegeneration in PD, suggesting that cell-intrinsic factors can gate selective vulnerability. The most vulnerable neuronal subpopulations include SNpc DA neurons; cholinergic neurons in the pedunculopontine nucleus, nucleus basalis of Meynert, and dorsal motor nucleus of vagus; noradrenergic neurons in the locus coeruleus; and serotoninergic neurons in the raphe nucleus [4, 13]. While the factors regulating regional heterogeneity in disease susceptibility are not fully known, mitochondrial stress and failure of mitochondrial quality control pathways are thought to contribute to regional differences in pathology and neurodegeneration [4]. Here, we review the contribution of mitochondrial dysfunction to selective neuronal vulnerability in PD and summarize the current understanding of neuronal mitochondria maintenance through PINK1/Parkin-mediated mitochondrial quality control.

## Main text

### Importance of mitochondria in PD

#### The many facets of mitochondrial function

Mitochondria are membrane-bound organelles that perform a diverse range of critical cellular functions. They are double-membraned structures, with outer and inner mitochondrial membranes (OMM and IMM respectively) separated by an intermembrane space and a central matrix enclosed by the IMM. Reflecting their evolutionary origins as endosymbiotic bacteria, mitochondria carry their own unique circular genome (mtDNA) at copy numbers upwards of 10–100 per mitochondrion [14]. Their genome encodes two unique rRNAs, 22 tRNAs, and 13 polypeptides required to assemble the mitochondrial ribosome and parts of the electron transport chain (ETC), while the nuclear genome encodes 1000+ mitochondrial genes [14, 15]. While traditional textbook pictures show mitochondria as static, bean-shaped structures, in reality they exist as dynamic networks shifting from innumerable punctate organelles to cell-wide tubular networks governed by a complex fission/fusion machinery [16]. Mitochondria are highly multifunctional. They not only generate the bulk of ATP in most cell types through oxidative phosphorylation, but also metabolize and synthesize complex macromolecules (e.g. lipids, amino acids, and nucleotides); buffer reactive oxygen species (ROS) and cytoplasmic Ca^2+^; regulate cellular redox balances; control apoptosis; and serve as key anchoring scaffolds for intracellular signaling networks [14, 17, 18].

Though critical for cell survival, these energetically demanding processes generate reactive intermediates and oxidizing agents that damage nucleic acids, proteins, and lipids, necessitating various waste removal and damage control mechanisms such as the urea cycle, glutathione antioxidants, and H_2_S detoxification [16–18]. While these mechanisms perform detoxification of reactive metabolic intermediaries and end products, mitochondria also possess several sophisticated systems for maintaining structural integrity and proper protein function. These systems, collectively known as mitochondrial quality control (MQC), include AAA proteases that degrade proteins in the matrix and intermembrane space, the ubiquitin-proteasome system for removing OMM proteins, the removal of larger portions of mitochondria through mitochondrial derived vesicles (MDVs) and mitophagy, and regulation of fission/fusion dynamics [15, 19].

#### Neuron subpopulation-specific bioenergetic vulnerabilities in PD

Mitochondria within the CNS exist in a unique metabolic environment due to the sheer energetic demand of neural activity and the structural polarization of CNS cells. The brain comprises roughly 2 % of total body mass yet consumes 20% of the body’s oxygen intake and 25% of glucose supply, of which the bulk goes towards sustaining membrane potentials and facilitating neurotransmission [19, 20]. Neuronal mitochondria must meet the immense energetic demands of neuronal signaling while also buffering waves of Ca^2+^ entry, which leads to the generation of excitotoxic ROS if left unchecked [20]. Furthermore, neuronal architecture is complex and exquisitely polarized, with some neurons carrying the vast majority of their cytoplasm and mitochondria in long dendrites and axons that can be as far as a meter away from the soma [15, 21]. Given the functional specialization of these cellular subcompartments, it is likely that such structural polarization leads to local metabolic needs that may require sub-specialization of mitochondrial function, such as increased Ca^2+^ buffering at the pre- and postsynaptic termini and increased biosynthetic functions at the soma. While other somatic cells can oftentimes rely on cell division to generate fresh mitochondria, neurons are postmitotic cells that must overcome the aforementioned challenges for an entire lifetime.

These bioenergetic demands are particularly evident in the neuronal populations selectively vulnerable in PD, including most of the nuclei described in the introduction. Many of these neurons send extensive, branching axons throughout the brain and influence large, diffuse brain areas [4]. The axons of SNpc DA neurons, for example, form vast branching nets that have been estimated to form up to 100–400 thousand synapses within the striatum and extend on average around 30–46 cm in length in rats [22–24]. In humans, they have been estimated to form up to 1–2 million synapses [15]. When compared to the less vulnerable neighboring DA neurons in the ventral tegmental area, SNpc DA neurons have more complex axons, a higher density of axonal mitochondria, higher rates of oxidative phosphorylation, and increased superoxide production [25]. Genetic perturbation of MQC in cultured SNpc DA neurons led to a reduction in the size of axonal arbors, while neurons with smaller axonal arbors tended to be more resilient to MPP+, providing further evidence that the morphological architecture of SNpc DA neuron axons imposes significant strain on mitochondrial function [26]. Furthermore, many of these vulnerable populations extend unmyelinated axons [21], which likely demand even more energy than myelinated neurons due to the need to regenerate the membrane potential along the entire axon rather than just at nodes of Ranvier. Thus, the extreme cytoarchitectural specialization of these neuronal subpopulations places a large bioenergetic burden on their mitochondria and may contribute to their selective vulnerability in PD.

#### Evidence of mitochondrial dysfunction in human PD

The idea that mitochondria may be involved in the pathogenesis of PD was first suggested by observing the effects of MPTP, a byproduct of illicit synthesis of the opioid drug desmethylprodine. MPTP is metabolized to the mitochondrial complex I inhibitor MPP+, which in turn causes acute-onset Parkinsonism with selective destruction of SNpc neurons [2, 27]. Since the discovery of MPTP, three major lines of evidence – epidemiological, pathological, and genetic – have pointed to mitochondrial dysfunction as a central driver of disease. First, mitochondrial toxins have either been shown to cause or correlate with increased risk of PD. In addition to MPTP, exposure to the pesticide rotenone, a complex I inhibitor, has been associated with increased risk of PD in epidemiological studies [28–30]. Second, postmortem studies of human PD patients have found widespread evidence of mitochondrial dysfunction. Complex I dysfunction has been consistently identified in the SNpc of deceased patients, with some reports of more generalized complex dysfunction that may affect other tissues as well [31–34]. In addition to these bioenergetic defects, mitochondria in postmortem tissue also show evidence of genetic defects, with greater age-dependent accumulation of mtDNA deletions and somatic mosaicism than control subjects [33–36]. Numerous other studies have identified dysregulation in the expression of various mitochondrial proteins, such as the molecular chaperone prohibitin, OMM protein VDAC1, mitochondrial import protein Tom40, and serine protease HtrA2 [37], as well as increased oxidative damage to mitochondrial proteins [38]. Third, mutations in genes that cause monogenic PD have been linked to mitochondrial function. For example, the PD-associated gene VPS35, which encodes a key subunit of the retromer complex responsible for sorting proteins between membranous organelles [39], contributes to the formation of MDVs and regulates fission/fusion dynamics [40–42]. The mutant proteins encoded by other genes that cause monogenic PD, such as *LRRK2, SNCA, ATP13A2*, have likewise been found to cause mitochondrial pathologies ranging from increased fragmentation, disruption of ER-mitochondrial interactions, impaired Ca^2+^ buffering, elevated numbers of mtDNA mutations, and increased ROS production [43–47].

**Fig. 1:**
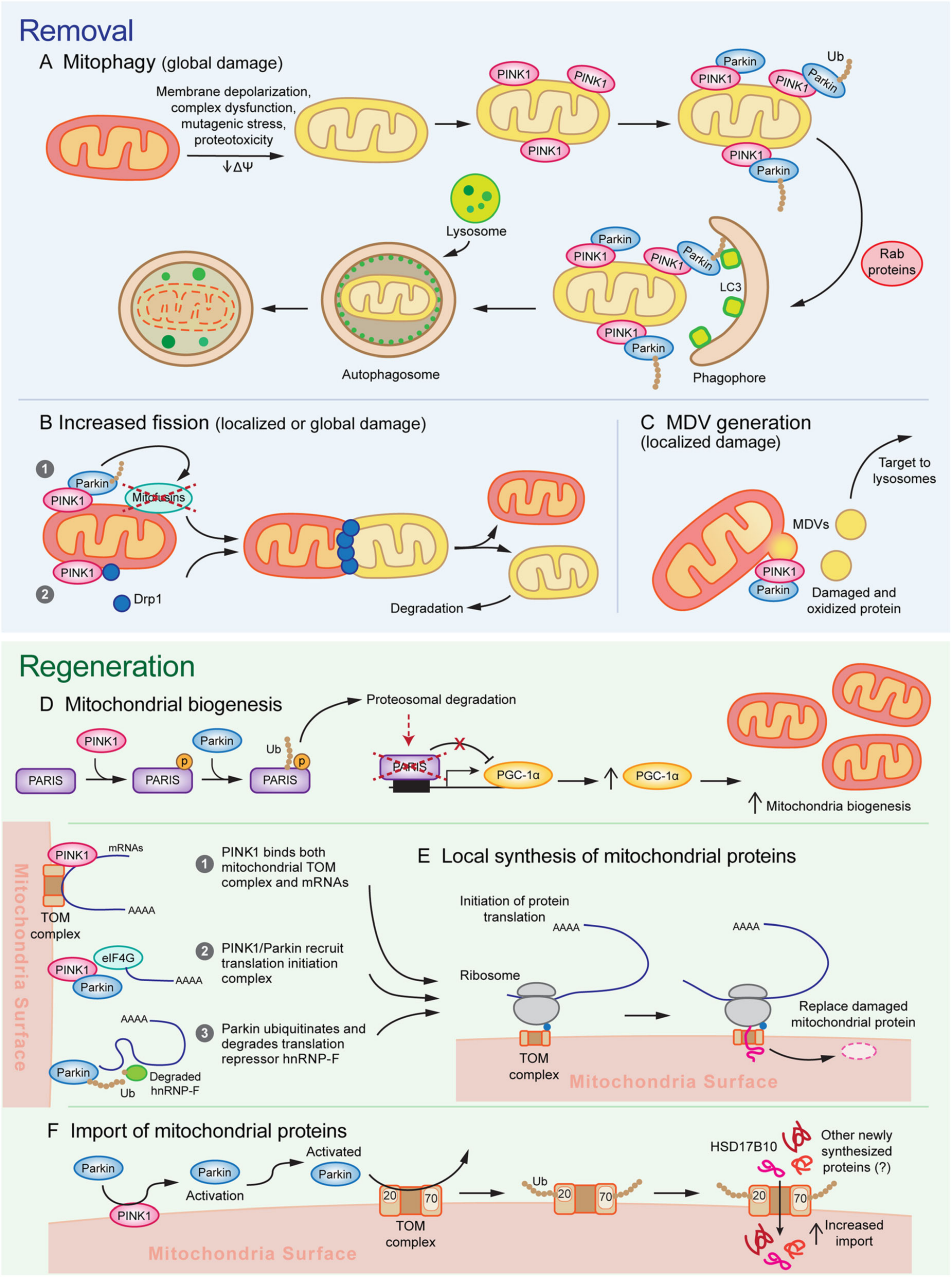
A model for the multifunctional role of PINK1/Parkin in mitochondrial quality control. Activation of PINK1/Parkin triggers multiple sequential and parallel mechanisms of **a-c** mitochondrial removal and **d**, **e** mitochondrial regeneration. Different mechanisms of mitochondrial removal are engaged depending on the severity of damage. **a** Mitochondria experiencing global/widespread damage undergo mitophagy, in which massive PINK1/Parkin activation recruits autophagosome membranes via Rab proteins and LC3 and is subsequently degraded by lysosomes, and **b** undergo mitochondrial fission caused by PINK1/Parkin dependent mitofusin degradation and Drp1 recruitment. **c** Focal damage leads to the activation of mitochondrial fission as well as mediate the Drp1-independent formation of MDVs, which allow for removal and destruction of small pockets of damaged mitochondrial components and limits the nonspecific destruction of functioning subdomains. **d** To replace the mitochondrial components removed through removal mechanisms, PINK1 phosphorylates PARIS and primes it for ubiquitination by Parkin. Subsequent proteosomal degradation of PARIS relieves PARIS-mediated transcriptional repression of PGC-1α, thereby stimulating mitochondrial biogenesis. **e** Furthermore, recent evidence suggests that PINK1/Parkin may promote local synthesis of nuclear-encoded mitochondrial proteins by bringing mRNAs encoding mitochondrial genes to the mitochondria and promoting translation initiation. **f** PINK1/Parkin activation further leads to the ubiquitination of TOM complex proteins Tom70 and Tom20, which promotes transport of newly synthesized proteins into the mitochondria, possibly as a means to facilitate the replacement of damaged protein degraded through other mechanisms

Most prominent of the monogenic PD-associated genes involved in mitochondrial function are *PINK1*, encoding the PTEN-induced serine/threonine kinase 1, and *PRKN*, encoding an E3 ubiquitin ligase Parkin [18, 33, 48, 49]. Following these findings, genetic studies in *Drosophila* implicated a shared biological pathway for Parkin and PINK1 function [50–52], with further mechanistic work establishing their function in detecting mitochondrial damage and recruiting mechanisms to remove and replace dysfunctional mitochondrial components. The activation and functions of the PINK1/Parkin system of MQC are arguably some of the most well-studied pathways of PD pathogenesis and will be reviewed in detail below (Fig. 1). Collectively, these findings firmly establish mitochondrial dysfunction as a core pathologic feature of PD. The contribution of mitochondrial dysfunction to neurodegeneration relative to other mechanisms is not fully known, though it likely differs between monogenic versus familial PD and is dependent on the brain region in question.

### PINK1/Parkin as core organizers of mitochondrial quality control

#### Mutations in *PINK1* or *PRKN* (Parkin) cause selective loss of SNpc DA neurons

Loss of function mutations in *PINK1* and *PRKN* are the most common known causes of autosomal recessive and early onset PD (before the age of 45) [48, 49, 53]. Despite an earlier age of onset, PD associated with *PINK1* or *PRKN* mutations is usually more benign with slower progression, high L-DOPA responsiveness, and normal cognition, but with high likelihood of dyskinesias, dystonia, hyperreflexia, and psychiatric symptoms [53–55]. The clinical presentation of *PINK1/PRKN* PD is intriguing in its relatively pure motor phenotype compared to other cases of PD and the robust and long-lasting (sometimes in the range of decades) responsiveness to dopamine replacement therapy, suggesting that these patients may experience a disease process that is largely confined to the SNpc DA system. This hypothesis is consistent with postmortem pathology in seventeen cases of *PRKN* and one case of *PINK1* PD, which is striking for the highly specific loss of SNpc neurons with relative sparing of the locus coeruleus (LC) and other brain regions [53, 56]. Whereas LB pathology is found in virtually all cases of sPD, it was found only inconsistently in *PINK1/PRKN* PD (6/17 genetically confirmed *PRKN* PD, and trace amounts in 1/1 *PINK1*), suggesting that α-synuclein may be a minor player in these cases [53, 56]. In contrast, the clinical-pathologic features of sporadic PD and other monogenic causes of PD tend to exhibit significantly greater variation and wider involvement of other cell populations [54–56]. While it is important to note that the limited availability of autopsy cases may cause an underestimation of the pathological heterogeneity of *PRKN* and *PINK1* PD*,* the combined clinical-pathological evidence of highly selective SNpc DA neuron loss suggests that these genes may represent an Achilles heel of SNpc DA neurons and that studying downstream pathological pathways may be critical for yielding insights into the vulnerability of the population in PD.

#### Mechanism of PINK1/Parkin activation

PINK1 and Parkin function as the first steps of a signaling pathway that activates mitochondrial quality control pathways in response to mitochondrial damage [57]. Under basal conditions, PINK1’s N-terminus is transferred across the OMM to the IMM, with the kinase domain located closer to the C-terminus protruding out into the cytosol. PINK1 is then cleaved by IMM-bound proteases and subsequently degraded by the proteasome, leading to undetectable basal levels of PINK1 [58, 59]. Stressors such as membrane depolarization, mitochondrial complex dysfunction, mutagenic stress, and proteotoxicity lead to accumulation of PINK1 on the OMM by impairing intermembrane transport of the N-terminus domain to the IMM. Subsequent homodimerization of PINK1 on the OMM leads to autophosphorylation, which promotes kinase activation and facilitates binding to substrates Parkin and ubiquitin [58–61]. Thus, PINK1’s ability to rapidly accumulate and activate in response to mitochondrial stressors allows it to function as a sensor of mitochondrial damage.

Parkin is an E3 ubiquitin ligase that contains a ubiquitin-like domain and four RING domains: RING0, RING1, IBR, and RING2 [62]. Its basal activity is minimal due to intramolecular interactions that block the active site and compete with E2 ligase binding [57]. Upon mitochondrial injury, PINK1 activates Parkin through two mechanisms. First, it phosphorylates ubiquitin on S65, which competes with an autoinhibitory domain within Parkin and stabilizes it in an active conformation. Second, PINK1 directly phosphorylates Parkin on S65 in Parkin’s ubiquitin-like domain, which induces conformational changes that allow for binding of the charged E2 ligase [57–59, 63–70]. These mechanisms increase Parkin’s E3 ubiquitin ligase activity, with both required for full activation, though it is unclear whether either mechanism contributes differentially to activation [63, 66, 71]. Thus, Parkin amplifies a damage detection signal from PINK1 by facilitating the formation of ubiquitin chains, which recruit more Parkin to the mitochondria [57].

Once recruited to the mitochondria, Parkin exhibits two waves of ubiquitination: the first wave targets many outer OMM and mitochondrial matrix proteins within the first 2 h of activation, whereas the second wave targets IMM proteins [58, 72, 73]. Parkin has also been found to ubiquitinate many cytosolic targets [73], though it is unclear whether these targets are phosphorylated by mitochondrial bound Parkin or whether there may be cytosolic activation of Parkin. Among these cytosolic targets are AIMP2, whose accumulation leads to PARP1 and MIF-dependent cell death of nigral DA neurons; and Parkin Interacting Substrate (PARIS, ZNF746), which causes neurotoxicity by suppressing mitochondrial biogenesis [74–82]. The diversity of Parkin substrates and the formation of multiple types of ubiquitin linkages (K6, K11, K48, and K63), poses the question of whether Parkin ubiquitination may have diverse effects on cellular signaling beyond targeting proteins for degradation [18, 73, 83]. Substrate specificity and chain formation may show some cell-type dependence. For example, a recent proteomic profile of Parkin substrates in HeLa cells and human neurons suggests differences in which substrates are targeted, which residues are ubiquitinated on the same substrate, and which ubiquitin chains are generated [84]. Thus, it is possible that differences in Parkin substrate recognition and targeting may lead to divergent Parkin function in a cell-type specific manner.

#### PINK1/Parkin regulate tiers of mitochondrial quality control pathways

##### Parkin/PINK1-mediated mitophagy

A prevailing model for PINK1/Parkin function in recent years has suggested that their accumulation on the outer mitochondrial membrane can trigger the macroautophagy of mitochondria (mitophagy) (Fig. 1a). In numerous cell lines including HeLa cells and mouse embryonic fibroblasts, exposure to numerous mitochondrial toxins such as CCCP, antimycin A, valinomycin, and rotenone reproducibly triggers the accumulation of PINK1 on the mitochondrial membrane and the subsequent recruitment of Parkin [59, 85–89]. Formation of ubiquitin chains on mitochondrial proteins leads to the binding of autophagy receptors, such as optineurin and NDP52, as well as Rab signaling proteins RABGEF1, RAB5, and RAB7A to the mitochondrial surface [90–93]. These mediators of mitophagy assemble autophagosomal membranes to eliminate damaged mitochondria [94]. While these findings have been robustly demonstrated in immortalized cell lines exposed to mitotoxic and mutagenic stress, compelling evidence of PINK1/Parkin-mediated mitophagy occurring in neurons in a disease-relevant context remains scarce.

Both in cultured neurons and in the mouse brain*,* mitochondrial translocation of PINK1/Parkin onto mitochondria is far less consistently observed or dramatic than that observed in cell lines. In comparison to cell lines, cultured primary neurons show much weaker recruitment of Parkin to mitochondrial surfaces, slower temporal kinetics on the order of ~ 6–18 h of exposure to CCCP, and oftentimes require a combination of exogenous Parkin overexpression and apoptosis inhibitors to maintain neuronal survival under these conditions [16, 18, 95–98]. Even in cases where neuronal parkin translocation was observed, most studies do not assay mitophagy directly, instead using proxy measures such as mitochondrial membrane potential or mtDNA levels that cannot rule contribution from other PINK1/Parkin functions (see below) [98, 99]. One important factor that may complicate the study of PINK1/Parkin mitophagy in neurons is subcellular localization and local factors may gate activation of this system. A recent study suggests that damage to axonal mitochondria in cultured neurons can induce rapid (within ~ 1 h) Parkin translocation followed by recruitment of autophagosomes and removal via lysosomes [100]. Thus, in contrast to cell lines, variability in the intracellular environment and mitochondrial stresses of different neuronal compartments may impose additional limitations to PINK1/Parkin mitophagy.

A few recent studies using mitochondria-targeted pH-sensitive fluorescent indicators suggest that low levels of basal mitophagy occur throughout both wild-type mouse and *Drosophila* brains, including in DA neurons [101–106]. The rate of basal mitophagy appears to increase with the metabolic demands of the tissue and in response to stressors such as hypoxia and mtDNA mutagenic stress [102, 104, 106]. Though age also appears to affect the rate of basal mitophagy, the directionality of the effect is unclear, as it has been reported to increase with age in *Drosophila* but decrease in mice [102, 106]. Despite evidence of mitophagy occurring in vivo, it remains unclear whether PINK1/Parkin activation plays the same role in basal mitophagy in vivo as it does in artificially induced mitophagy in vitro. A proteomics study quantifying rates of protein turnover in *Drosophila* via stable isotope labeling found that Parkin or autophagy-deficient Atg7 mutants showing increased half-lives in mitochondrial proteins [107]. However, the effects on mitochondrial protein half-lives were only weakly correlated between the two mutants, suggesting either that mitophagy may be influenced by Parkin-independent regulatory pathways or that Parkin may regulate mitochondrial protein turnover through autophagy-independent mechanisms [107]. Moreover, three of the previously mentioned studies found that PINK1 and Parkin knockouts had no effect on basal mitophagy in mouse and *Drosophila* [103–105], whereas one study found that PINK1 or Parkin deficiency in *Drosophila* abrogated an age-dependent increase in basal mitophagy [106]. Furthermore, if driving mitophagy is a primary role of endogenous Parkin/PINK1, then one might expect that loss of either gene would lead to accumulation of mitochondria. However, numerous reports have found that PINK1-KO or Parkin-KO lead to reductions in mitochondrial content in neurons in vitro and in vivo [80, 95, 108]. Thus, there is yet to be a convincing demonstration that PINK1/Parkin play a major role in driving mitophagy in the mature CNS. This does not rule out the possibility that PINK1/Parkin-independent mitophagy pathways play a role in the CNS (reviewed in [109]), as many of these pathways showing cell type and context-dependent activation. Because most of these potential alternative mitophagy pathways were studied in non-neuronal cell lines and tissues [109], further experimentation is required to establish their role in the nervous system.

A central question that remains is why PINK1/Parkin mitophagy is readily inducible in cell culture yet difficult to observe in neurons. One possibility is that PINK1/Parkin mitophagy may be gated by insult-specific or localization-specific factors. For example, it is possible that only certain sources of mitochondrial stress may trigger PINK1/Parkin activation, such as accumulation of mtDNA mutations by knocking out a mtDNA repair gene [96]. Though mtDNA mutations do accumulate in human PD cases, it is unclear how accurately mutant mice recapitulate the stress experienced in the human disease and whether mtDNA mutations cause or simply correlate with disease. In addition, some findings have suggested that PINK1/Parkin mitophagy occurs preferentially in the distal axons [100, 110], where turnover of defective mitochondria may be more frequent due to the difficulty of maintaining mitochondria so far from the soma. Presynaptic mitochondria can sometimes take days to be trafficked from the soma to their destination [15], during which time mitochondrial proteins are damaged by ROS produced through oxidative phosphorylation and undergo normal turnover. Compared to mitochondria at the soma, synaptic mitochondria tend to undergo increased oxidation during aging, show higher vulnerability to Ca^2+^-induced damage, and exhibit lower spare respiratory capacity [19, 111–113]. Given the damage dependence of mitophagy, it is possible that synaptic/axonal mitochondria may rely more on this process for ensuring MQC. These possibilities require careful exploration of physiologically relevant mitochondrial insults and more precise subcellular localization of PINK1/Parkin in order to establish their true role in mitophagy.

It is also possible that the bioenergetic demands of neurons are incompatible with significant upregulation of mitophagy. Immortalized cell lines are highly glycolytic due to their origin as cancer cells and thus can likely afford to degrade many of their mitochondria. However, neurons rely primarily on oxidative phosphorylation to survive, generating nearly 95% of their ATP through oxidative phosphorylation [16, 18–20]. Even under acute mitochondrial injury, neurons cannot switch a substantial portion of ATP generation from oxidative phosphorylation to glycolysis [97]. Indeed, forcing HeLa or RPE1 cells to rely on oxidative phosphorylation through the use of galactose as the main carbohydrate source greatly inhibits stress-induced Parkin translocation and blocks mitophagy [97, 114]. Thus, while it is possible neurons undergo a low level of basal mitophagy, their dependence on oxidative phosphorylation and the sparsity of mitochondria in distal neuronal processes may prevent neurons from undergoing extreme injury-induced PINK1/Parkin-mediated mitophagy as observed in cell culture.

##### Mitochondrial QC through fission/fusion regulation

Mitochondrial networks undergo constant remodeling and depend on the balance between fission and fusion to meet the changing metabolic needs of their host cell. For example, increased mitochondria fission leads to the generation of smaller mitochondria, which facilitates intracellular transport (such as distribution of mitochondria into the dendrites of Purkinje neurons), heat generation in brown adipose tissue, mitophagy, and apoptosis [115–117]. Conversely, enhanced fusion increases mitochondrial length, which may increase the efficiency of oxidative phosphorylation and allow cells to meet higher energy demands [115, 116]. In mammals, the primary mediators of fission and fusion are dynamin family proteins. Fission requires a single protein, Drp1, which assembles into multimeric spirals around mitochondrial tubules and constricts them in a GTP-dependent fashion [115, 116]. Fusion is more mechanistically complex and requires mediators on the OMM (Mitofusins 1 and 2) and IMM (Opa1) [115, 116]. In response to different stressors, fission/fusion dynamics undergo one of two responses. Mild stress such as nutrient deprivation and mild toxin exposure leads to stress-induced mitochondrial hyperfusion, possibly facilitating the combination of mildly damaged mitochondrial components with healthy ones to dilute the effects of damage. More severe damage such as mitochondrial depolarization promotes fission, leading to the formation of smaller fragments that can undergo mitophagy or other forms of removal [115]. However, it is unclear how this stress response varies across cell types, how cells decide to undergo hyperfusion or fragmentation, and what mechanism(s) lead(s) to the two different responses.

Early studies characterizing Parkin- or PINK1-null *Drosophila* mutants found evidence of swollen mitochondria in numerous tissue [50–52, 118, 119], suggesting that PINK1 and Parkin may either drive fission or inhibit fusion. These pathological features could be ameliorated by increasing expression of Drp1 or reducing Opa1 or Mitofusin [120–123], suggesting a genetic interaction between PINK1/Parkin and canonical fission/fusion regulatory pathways. Both PINK1 and Parkin promote degradation of critical mitochondrial fusion proteins Mitofusin 1 and 2 [72, 124–131], while PINK1 is sufficient to promote mitochondrial fission by recruiting Drp1 to mitochondria [132, 133]. Thus, Parkin/PINK1 activation seems to drive mitochondrial dynamics towards fission by activating pro-fission and inactivating pro-fusion pathways (Fig. 1b). PINK1/Parkin-induced mitochondrial fission potentially contributes to MQC through two parallel mechanisms. First, it could act to segregate areas of focal damage. For example, a recent study in HeLa cells used mutant, aggregation-prone ornithine transcarbamylase (OTC) to induce misfolded protein foci in mitochondria [134]. These foci led to local accumulation of PINK1/Parkin and subsequent OTC clearance by mitochondrial fission [134]. Ablating mitochondrial fission through Drp1-KO did not affect the rate of OTC clearance; instead, it lead to generalized recruitment of PINK1/Parkin and substantial upregulation of mitophagy [134]. Thus, in the case of focal damage, mitochondrial fission appears to be an initial defense which enables selective removal of dysfunctional components, thereby preventing mitophagy from destroying healthy mitochondria. However, this process relies on focal concentrations of mitochondrial damage. It is unclear if and how mitochondrial fission could contribute to the management of more diffuse damage or to baseline MQC in the absence of an insult.

A second potential mechanism for fission-regulated MQC relies on the ability of fission/fusion cycles to locally enrich for degradation targets. A proteomic profile of stationary phase yeast found heterogeneity in the turnover rates of different mitochondrial matrix proteins, and that deleting Dnm1 (yeast Drp1) suppresses some of these differences [135]. These findings suggest that during baseline MQC, mitochondrial fission may engage a selectivity filter that enriches for certain degradation targets and segregates them for replacement. While the nature of this selectivity filter is still largely unknown, a recent study has suggested that differential phosphorylation on substrates may contribute to target selection [136].

##### Removal of focal damage through mitochondrial derived vesicles

While mitochondrial fission may be well-suited for the segregation of relatively large domains of damaged mitochondrial components, it may lead to unwanted elimination of healthy regions when damage is confined to smaller domains. A potential mechanism allowing for more selective isolation of damaged components is the formation of MDVs, which have a diameter of 70–150 nm and can be either single or double membraned [137]. The formation of MDVs is Drp1-independent, indicating that it is mechanistically distinct from mitochondrial fission [138, 139]. A number of subtypes of MDVs have been identified, including those that traffic to lysosomes, multivesicular bodies, macrophage phagosomes, and peroxisomes [137, 140–142]. Numerous markers have been found to mark MDVs including Tom20, MAPL, Stx17, Pex3, Rab7/9, and Sod2 [137, 140–143]. The presence of specific proteins on different MDV subpopulations seems to contribute to differential end targets. For example, MAPL+ or Pex3/Pex14+ MDVs target peroxisomes, Sod2+ MDVs target bacteria-containing phagolysosomes, and Stx17+ or Tom20+ MDVs are trafficked to the endolysosomes [137–139, 141–144]. However, the overlap in marker expression between MDV subtypes and the precise correlation between markers and MDV functions or targeting properties are not yet fully elucidated. While molecular and functional profiling of different MDV subtypes is still in its early stages, MDVs are emerging as potential facilitators of mitochondrial quality control [137], peroxisome biogenesis [142], and immune function [140, 141, 145]. While it has been demonstrated that PINK1/Parkin are involved in the generation of some MDVs, such as those destined for endolysosomes and those involved in destruction of endocytosed bacteria in macrophages [137, 141], it is not clear whether all MDVs are generated via PINK1/Parkin activation. MDVs containing only OMM proteins such as Tom20 may not require PINK1/Parkin [144], suggesting that certain subpopulations of MDVs may be PINK1/Parkin independent.

One important subgroup of PINK1/Parkin-dependent MDVs may preserve mitochondrial integrity by removing localized patches of mitochondrial damage (Fig. 1c). Production of these MDVs is stimulated by the presence of oxidative and mutagenic stress and their contents tend to be enriched with oxidized proteins [138, 144, 146]. Incorporation of protein cargo into these MDVs is dependent on PINK1/Parkin and shows a degree of cargo selectivity dependent on the nature of the insult. For example, cytosolic ROS generated from xanthine oxidase leads to the incorporation of OMM-localized VDAC, whereas mitochondrial ROS resulting from antimycin A lead to incorporation of IMM-localized complex III [139, 144]. Ultimately, the subpopulation of MDVs enriched for oxidized protein cargo are targeted to the lysosome for degradation [138, 144]. Furthermore, Parkin-null or PINK1-null *Drosophila* mutants show increased half-lives of mitochondrial proteins, with a selective overrepresentation of ETC components [107]. Given that ETC components are exposed to a highly oxidative environment, one intriguing possibility – as pointed out by the authors – is that PINK1/Parkin-associated MDVs may provide a vehicle by which oxidatively damaged ETC components are selected for turnover. Overall, it is likely that MDV formation is an earlier and milder response to stress compared to mitophagy. The temporal progression of MDV formation supports this hypothesis, as antimycin A, oligomycin, or CCCP causes MDV formation in HeLa cells with the peak rate occurring on the order of 1–4 h, whereas mitophagy occurs on the scale of 4–24 h [139].

Whereas mitophagy is a more severe response to stress resulting in the destruction of entire mitochondria, MDVs may play an intermediate role contributing to mitochondrial homeostasis. MDV formation has also been shown to occur under baseline conditions in cell lines and cardiomyocytes [138, 139, 146], with varying percentages of mitochondrial proteins (roughly 1–4%) being ejected via MDVs in a cell-free mitochondrial budding assay [144]. These findings suggest that PINK1/Parkin-mediated MDV formation may serve as a mechanism for selectively isolating and eliminating pockets of damaged mitochondria while preserving the integrity of the remaining mitochondrial network, and thus may be preferable to mitophagy for neurons. In support of this hypothesis, mitochondria in cardiomyocytes, which likewise depend on oxidative phosphorylation, have been shown to form MDVs both at a basal rate under resting conditions and at a heightened rate when under mutagenic stress from the chemotherapeutic doxorubicin [146]. While this study also found evidence of highly limited mitophagy when cardiomyocytes were exposed to doxorubicin, the number of MDV budding events outnumbered mitophagosome formation by an order of magnitude. These findings in heart tissue provide evidence for a mechanism by which cells reliant on oxidative phosphorylation can use MDVs as a physiological system for MQC. However, further validation is needed to confirm that MDVs serve the same function in the nervous system.

#### PINK1/Parkin facilitate the generation of new mitochondrial components

##### Stimulation of mitochondrial biogenesis through PGC-1α

While removal of damaged mitochondria is crucial in order to limit the consequences of injury, it is equally important to generate new mitochondria as replacement. Mitochondrial biogenesis is governed in large part by the PGC-1 family of transcription factors, including PGC-1α, PGC-1β, and PRC. PGC-1 family proteins regulate numerous downstream targets, including transcription factors Nrf-1 and Nrf-2, which subsequently increase cellular respiration rates, energy utilization, and mitochondrial biogenesis (see review [147]). PINK1/Parkin regulate PGC-1α activation through degradation of PARIS. PARIS is a KRAB and zinc finger protein that binds to an insulin responsive sequence of PGC-1α and induces its transcriptional repression [79]. PINK1 directly phosphorylates PARIS at S322 and S613, priming it for ubiquitination by Parkin, which interacts with the C-terminus zinc finger of PARIS and tags it for destruction [79–81, 148]. Loss or inactivation of Parkin leads to accumulation of PARIS, which downregulates PGC-1α, leading to selective degeneration of SNpc DA neurons that can be rescued by overexpressing PGC-1α and restoring mitochondrial biogenesis [79, 80, 82]. Conversely, overexpressing Parkin in WT cortical neurons increases PGC-1α levels, mtDNA copy number, and mitochondrial density [149]. Thus, by tagging PARIS for destruction, PINK1/Parkin drive the generation of new mitochondria by increasing PGC-1α levels (Fig. 1d).

These findings suggest that the role of the PINK1/Parkin system in mitochondrial turnover is intrinsically linked to its role in biogenesis. This raises an important question: why is a single system responsible for coordinating both biogenesis and mitophagy? One explanation is that the simultaneous activation of mitochondrial removal and biogenesis moderates changes in overall mitochondrial function; runaway activation of one process would have deleterious effects on cellular health. In support of this, while PGC-1α overexpression protects against insults such as Parkin loss, PARIS overexpression, α-synuclein overexpression, and rotenone [79, 80, 150, 151], its overexpression in the SNpc of WT mice leads to loss of DA neurons and increased sensitivity to MPTP [149, 152, 153]. Conversely, knocking out PGC-1α in mice potentiates sensitivity to MPTP toxicity, triggers formation of α-synuclein aggregates, induces gene expression changes consistent with those found in preclinical and early stage PD patient brains, and causes vacuolization in different brain regions during development [150, 154–157]. Conditional adult and cell type specific PGC-1α-KO likewise indicate that PGC-1α is necessary for the survival of SNpc DA neurons and basal ganglia medium spiny neurons, which are involved in Huntington’s disease [158, 159]. These findings indicate that mitochondrial density and energy production are highly dosage-sensitive. Thus, it may be advantageous for neurons to use PINK1/Parkin to as a central coordinator of both MQC and biogenesis rather than engaging two independent systems that may become unsynchronized in disease.

##### Local mitochondrial repair mechanisms through localized translation and protein import

PINK1/Parkin-mediated mitochondrial biogenesis acts primarily on the transcriptional level and acts to generate new, whole mitochondria. However, in cases of mild damage or basal MQC, it may be sufficient to replace individual proteins or protein complexes rather than entire mitochondria. Moreover, due to the dependence on transcription as a key regulatory step, biogenesis may not act quickly enough to sustain mitochondrial function in the event of acute damage, especially in distal processes far removed from the nucleus. One mechanism by which PINK1/Parkin may compensate for the limitations of PGC-1α-dependent biogenesis is by promoting localized translation of nuclear-encoded mitochondrial RNAs (nc-mtRNAs) on the mitochondrial surface. Organelle-localized translation has been most classically studied at the surface of the rough endoplasmic reticulum (RER), where secreted, plasma membrane, and other membranous organellar proteins are synthesized. Nuclear-encoded mitochondrial proteins are traditionally believed to be translated in the cytosol and then carried in an unfolded state by chaperones to the mitochondria, due to the presence of a mitochondrial targeting sequence (MTS) at the N-terminus of the preprotein [160, 161]. However, extensive differential centrifugation, IF colocalization, ultrastructure, and cross-linking experiments have established that a significant complement of nc-mtRNAs, up to 70% in yeast, are localized to and translated near the mitochondria [160–163]. Cytosolic ribosomes initiate translation of nc-mtRNA and bind to the mitochondrial translocase of the outer mitochondrial membrane (TOM) complex, which imports proteins into the mitochondria, via the nascent peptide chain in a mechanism reminiscent of the canonical synthesis of secreted and membrane proteins at the RER [160, 161]. Mitochondria-localized translation has been mostly studied in yeast and the extent to which this system is preserved in humans has not been thoroughly characterized yet. However, there are reports of specific genes encoding Oxa1, F1B-ATPase, TMEM126A that are translated at the mitochondria in human cell lines [160].

Recent studies have suggested that PINK1 and Parkin may play a role in facilitating localized translation of nc-mtRNAs. Loss of PINK1 in *Drosophila* neuromuscular tissue, human cell lines, and human DA neurons derived from patient iPSCs impairs localization of nc-mtRNAs to mitochondria, including several key ETC components, without affecting total RNA levels of these nc-mtRNAs [164]. Further genetic and biochemical studies described several potential mechanisms by which PINK1/Parkin could affect translation of mitochondria-localized nc-mtRNAs, including PINK1 serving as a binding scaffold for bringing together import receptor Tom20 and mRNA 5′ caps, PINK1/Parkin recruiting the translation initiation complex, and Parkin ubiquitinating and triggering degradation of translational repressor hnRNP-F [164]. These findings suggest that local recruitment of PINK1/Parkin on to mitochondria may induce the synthesis of new mitochondrial proteins, perhaps to replace damaged components removed through mitophagy or MDVs (Fig. 1e). Such a system of localized translation may be particularly advantageous in neurons as it reduces the costs of transport and potential protein misfolding errors from synthesizing proteins exclusively at the soma. For example, a recent study in cultured superior cervical ganglion axons found evidence of transcripts from at least 100 unique nuclear mtRNA genes localized to the axon [165], while an in vivo experiment pulling down ribosome-associated mRNA from retinal ganglion cell axons found enrichment of nuclear genes involved in mitochondrial function such as ETC components in the axonal compartment [166]. Given the unique architecture and bioenergetic demands of SNpc DA neurons, where the time needed to transport mitochondria to the presynapse may exceed the half-lives of many mitochondrial proteins [15], localized translation of nc-mtRNA is likely necessary to maintain mitochondrial function in a temporally and energetically efficient manner.

Furthermore, these nuclear encoded mitochondrial proteins rely on the TOM complex to enter the mitochondria, a process that recent findings suggest may be regulated by PINK1/Parkin (Fig. 1f). Several proteomics profiling and biochemical studies in *Drosophila* and human cells have found that PINK1-activated Parkin can induce ubiquitylation of TOM receptor proteins Tom70 and Tom20 [63, 72, 167, 168]. This ubiquitylation may increase the import of the endogenous mitochondrial protein HSD17B10 or a reporter peptide carrying a MTS, with cells carrying PD-associated *PINK1* or *PRKN* showing impaired import [168, 169]. If these findings generalize to other imported proteins, it would suggest that PINK1/Parkin may also directly drive protein influx into the mitochondria through posttranslational regulation of the TOM complex. While exciting, these preliminary findings require further validation in more complex, in vivo mammalian systems. Moreover, further studies into whether ubiquitylation always leads to increased import (vs. degradation), and whether this mechanism leads to global increases in protein import or may have target-specific effects (eg. on ETC components) may yield important insights into the contribution of PINK1/Parkin to mitochondrial protein import. Given that PINK1 stabilization serves as a sentinel signal for import defects [58, 59], the ability of PINK1/Parkin to subsequently promote mitochondrial import may act as a direct negative feedback mechanism to preserve function by ensuring a steady supply of fresh, undamaged protein components.

These exciting recent findings point to a novel role of PINK1/Parkin in driving the local supply and replacement of mitochondrial proteins without the need to rely on slow transcriptional processes in a distant nucleus. While much work lies ahead to establish the role of PINK1/Parkin in driving localized translation and protein import in both healthy and disease contexts in the mammalian CNS, these mechanisms hint at a degree of temporal and spatial flexibility in the PINK1/Parkin system in mitochondrial regeneration that has previously been underappreciated.

### Open questions for MQC in PD

#### Contribution of PINK1/Parkin MQC dysfunction to sporadic PD

While it is clear that genetic loss of PINK1/Parkin contribute to selective loss of SNpc neurons, these genetic cases represent only a small fraction of PD, which remains by and large a sporadic disease with no clear genetic etiology [1, 53]. Though plenty of evidence indicate that mitochondria dysfunction is widespread in sporadic PD cases (summarized above), these alterations are not necessarily specific to dysfunction of PINK1/Parkin MQC. Thus, the question of whether the mechanisms of MQC failure delineated in genetic models of PINK1/Parkin loss translate to the sporadic disease carries major implications for how we understand neuronal vulnerability in sPD.

Our understanding of MQC dysfunction in sPD has arisen largely from surveys of pathological changes in postmortem patient brains and mechanistic studies linking α-synuclein aggregation to deficits in the PINK1/Parkin pathway. The two broad classes of potential responses that PINK1/Parkin MQC may exhibit in sPD are either protective activation in response to mitochondrial damage, or inactivation leading to an additional pathway of neurodegeneration. While there is evidence that PINK1 levels are stabilized and increased in PD patient brains [170], Parkin is *S*-nitrosylated and sequestered into LBs, leading to reduced availability of soluble Parkin to perform its native functions [171–175]. Because Parkin acts downstream of PINK1 activation, its inactivation in sPD likely blocks the effects of PINK1 accumulation. This is supported by the accumulation of proteins normally targeted for degradation by the PINK1/Parkin system. Consistent with findings of Parkin inactivation in PD brains, protein levels of multiple Parkin substrates – AIMP2, FBP1, PARIS, PDCD2, STEP61 – have been found to be elevated in patient midbrain tissue [74–76, 79, 176, 177]. PGC-1α, whose levels would be expected to drop with inactivation of the PINK1/Parkin pathway, has likewise been found to be downregulated in PD brains [150, 155].

**Fig. 2:**
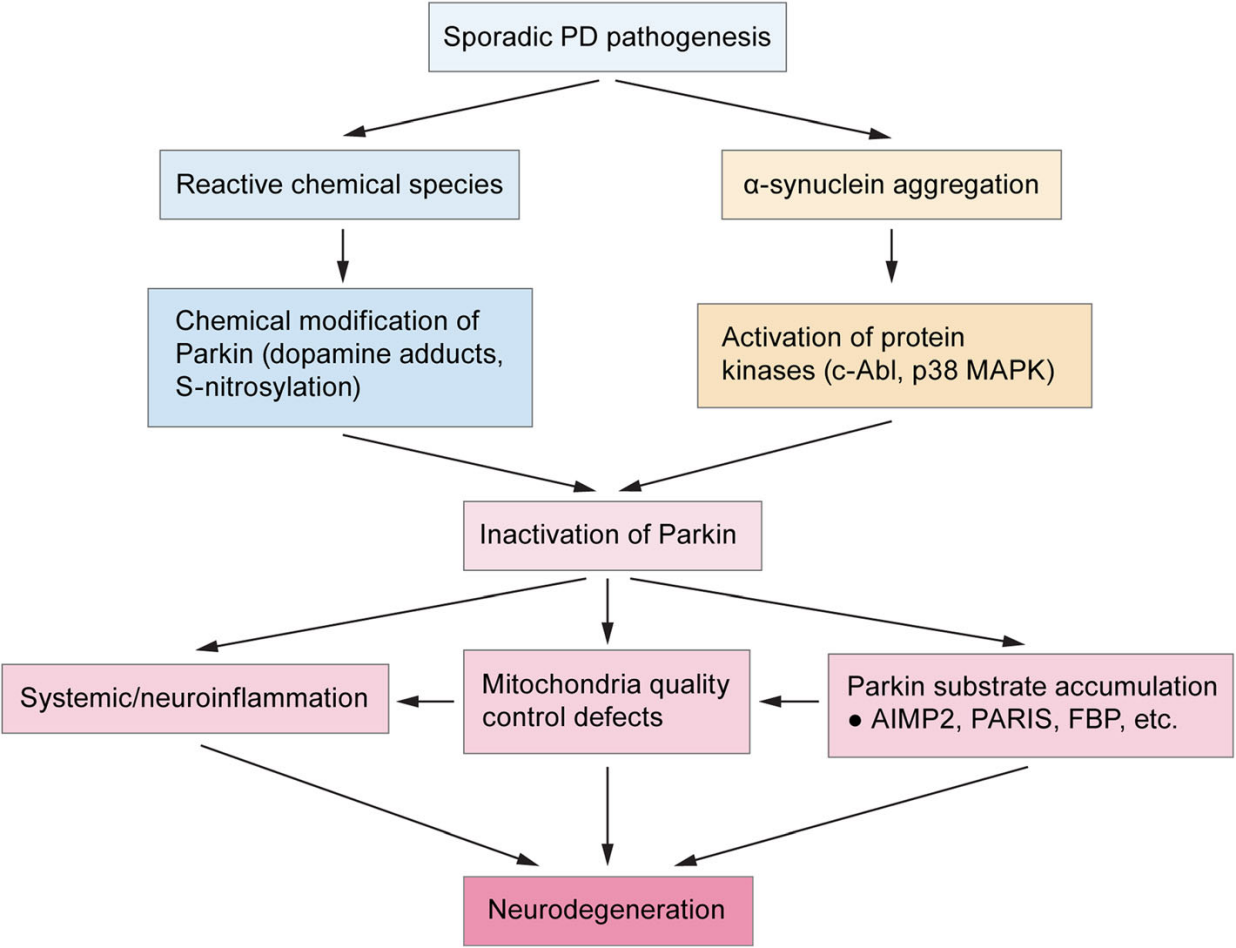
Mechanisms of Parkin inactivation in sporadic PD. To date, the two most widely studied mechanisms by which Parkin is inactivated in sporadic PD is through chemical modifications leading to impaired enzyme activity (dopamine adducts, *S*-nitrosylation), and through α-synuclein aggregation. α-Synuclein aggregates lead to the activation of stress-induced kinases c-Abl and p38 MAPK, which phosphorylate and inactivate Parkin. These mechanisms therefore suggest that studies on the molecular mechanisms of neurodegeneration caused by complete Parkin loss (ie. Genetic knockout) may also be common mechanism of neurodegeneration between Parkin-induced PD and sporadic PD of unclear etiology. Some of these downstream pathways of neurodegeneration include (neuro)-immune overactivation, mitochondrial deficits, and the accumulation of Parkin substrates leading to activation of cell-death pathways

Evidence that the PINK1/Parkin pathway is inactivated in PD raises two important questions: how does the pathway become inactivated in sPD, and to what degree does inactivation contribute to neurodegeneration? Current literature suggests that Parkin inactivation may derive from chemical inactivation [172, 174, 175, 178–180], or may occur downstream of α-synuclein aggregation (Fig. 2) [181, 182]. Initial findings that Parkin and α-synuclein do not directly interact with one another and that Parkin-KO failed to aggravate mutant α-synuclein toxicity in mice suggested that these proteins acted along independent pathways [183–185], though Parkin overexpression was able to rescue α-synuclein toxicity in multiple model systems [186–189]. However, these apparently contradictory results are consistent with a model in which Parkin inactivation occurs downstream of α-synuclein toxicity. A number of recent studies have suggested that α-synuclein pathology drives the activation of nonreceptor tyrosine kinase c-Abl, which phosphorylates Parkin at Y143 and inactivates it, leading to accumulation of Parkin substrates such as PARIS [75, 76, 181, 182, 190, 191]. Other potential mechanisms could involve direct sequestration of Parkin into α-synuclein aggregates [192] or the activation of other pathways that add inactivating posttranslational modifications on Parkin [193]. Thus, a possible explanation for why genetic Parkin-KO does not exacerbate α-synuclein toxicity is because it phenocopies the Parkin inactivation induced by α-synuclein.

An important caveat to many of these studies is the use of transgenic mice that overexpress mutant α-synuclein, indicating that these mechanisms must be validated in a disease model more representative of the human disease. One recent study from our lab attempted to bridge this gap in knowledge by using the preformed fibrils (PFF) model, a more physiologically accurate model system of induced α-synuclein pathology in WT mice. We found that blocking the accumulation of Parkin substrate PARIS rescued behavioral, molecular, and lifespan deficits as effectively as in transgenic α-synuclein mouse models [181]. Though these findings suggest that these mechanisms of α-synuclein-induced inactivation of Parkin are conserved from transgenic mouse models to human PD, more systematic efforts in non-transgenic model systems are needed. Furthermore, while these studies heavily emphasize the role of α-synuclein aggregation in inactivating Parkin (Fig. 2), whether α-synuclein-independent pathways of MQC inactivation such as oxidative damage may also be at play requires future study. Regardless, these findings implicate PINK1/Parkin inactivation not just as a cause of selective SNpc degeneration in the small percentage of PD cases associated with monogenic *PRKN* or *PINK1* mutations, but also in the sporadic disease driven by α-synuclein aggregation as well.

#### Mitochondrial dysfunction and neuroinflammation

While significant work has gone into understanding mitochondrial dysfunction within SNpc neurons in PD, it is less well understood how mitochondrial defects may contribute to neurodegeneration through non-cell autonomous mechanisms. While it is becoming increasingly clear that glial dysfunction and neuroinflammation play an important role in neurodegeneration in PD [194], the degree to which defects in MQC function contribute to neuroinflammation is relatively understudied. A few studies suggest that loss of PINK1 or Parkin may alter glial proliferation, leading to hypersensitized astrocytes and microglia that have greater levels of basal and triggered inflammatory cytokine release, nitric oxide (NO) production, and NLRP3 inflammasome activation [26, 195–198]. Co-culturing WT glia with Parkin-KO SNpc DA neurons has been shown to rescue neuron death and sensitivity to MPP+ observed in pure Parkin-KO co-cultures, suggesting that Parkin deficient glia contributes to cell death [26].

In addition to alterations in the glial inflammatory profile, compromised PINK1/Parkin MQC may also lead to pathological alterations to the interaction between the CNS and peripheral immune system. For example, PINK1/Parkin may play a role in suppressing mitochondrial antigen presentation (mitAP) on MHC-I in macrophages and dendritic cells [140, 145]. Postmortem human catecholaminergic neurons as well as cultured mouse SNpc neurons can express MHC-I receptors, which can be up-regulated over a PINK1-KO background or in response to infection, inflammatory mediators, oxidative stress, and α-synuclein [145, 199]. MitAP caused by loss of PINK1 can lead to brain infiltration of mitochondrial antigen-specific CD8+ cytotoxic T cells, which then attack SNpc DA neurons [145]. Thus, loss of PINK1/Parkin activity could trigger an adaptive immune response against mitochondrial proteins and engage the peripheral immune system in an improper assault against the CNS. Furthermore, these mechanisms may be occurring in a broader milieu of peripheral immune dysfunction. In macrophages, PINK1/Parkin generate MDVs containing mitochondrial ROS that are delivered to bacteria-containing phagosomes [141]. Loss of Parkin impairs bactericidal activity and leads to defective infection clearance, prolonged infection course, and elevated cytokine production [141]. Furthermore, human subjects with biallelic loss of Parkin show elevated systemic cytokine levels, with milder increases observed in heterozygous subjects [200].

These findings suggest that defects in MQC lead to three interesting effects on immune function that could contribute to PD neurodegeneration: an aggravated glia inflammatory phenotype, loss of immune tolerance and possible autoimmunity against neurons vulnerable in PD, and peripheral immune dysfunction. However, these mechanisms have largely been studied independently of one another. We lack an integrated model of how MQC defects produce (neuro)immune dysfunction and subsequent neurodegeneration. Furthermore, these mechanisms have been demonstrated in the context of global PINK1/Parkin ablation, whereas MQC defects in sPD may lead to more CNS-specific and milder immune dysfunction. Findings in sporadic PD patients of elevated systemic cytokines, CNS immune cell infiltration, and T cells recognizing α-synuclein peptide do suggest a certain degree of concurrent CNS and peripheral immune activation [194, 200, 201], but these studies are inherently correlative and give limited insight into the mechanisms by which these phenotypes arise. The degree to which diverse inflammatory mechanisms converge to cause neurodegeneration, and the importance of MQC defects to these mechanisms in sPD, are important areas of future research.

## Conclusions

### Cell-intrinsic and non-cell autonomous mechanisms

Early studies have long implicated selective vulnerability of SNpc DA neurons and mitochondrial dysfunction as core features of PD. While we now understand that PD processes are far more distributed across the CNS and may be driven primarily by prion-like mechanisms spreading α-synuclein aggregates, these non-cell autonomous mechanisms likely act in concert with cell- and region-specific factors that lead to selective vulnerability to neurodegeneration. Though these cell-intrinsic factors are likely complex and varied across the different vulnerable subpopulations, in SNpc DA neurons findings over the last few decades point to the unique mitochondrial challenges and stresses due to complex cytoarchitecture as a potential major cause. Probing the function of PINK1/Parkin has led to critical insights into their role in maintaining mitochondrial integrity and proteostasis in the face of the stressors faced by mitochondria. These protective mechanisms comprise multiple tiers of MQC, such as facilitating mitophagy, regulating fission/fusion dynamics, triggering removal of damaged mitochondrial components through MDV generation, promoting mitochondrial biogenesis by increasing PGC-1α, and regulating the local translation of mitochondrial genes (Fig. 1), though many of these proposed mechanisms require convincing validation in the mammalian CNS. Newer areas of research have begun to establish mechanisms by which α-synuclein aggregation causes inactivation of MQC (Fig. 2), which have clear implications for sPD, as well as how MQC defects in neurons and non-neuronal cells may contribute to neuroimmune mechanisms of neurodegeneration.

### Critical gaps in understanding

Despite the immense progress we have made, critical gaps in our understanding of PINK1/Parkin MQC remain. Our understanding of PINK1/Parkin pathways has been built up across a staggering variety of model systems ranging from *Drosophila*, *C. elegans*, mice, immortalized human cell lines, human iPSCs; whether all these pathways or a specific subset of these pathways is critical to the survival of human SNpc DA neurons requires further disambiguation. We have only just begun to elucidate the organizational principles of these diverse mechanisms, and it is likely that subcellular localization, cell-type specific factors, degree of damage, and nature of the damage are important factors governing which MQC processes become activated and when. For example, it is likely that MDVs activated in response to focal damage whereas mitophagy may be required for more severe, global mitochondrial damage. An additional possibility is that subcellular localization may shape dependence on PGC-1α-mediated biogenesis, a nucleus-dependent process, versus more spatially restricted mechanisms such as localized translation, which can operate in compartments far from the nucleus. Establishing the driving principles underlying how PINK1/Parkin juggle these various processes may answer many of the fundamental questions about cell-type vulnerability and disease mechanism in sPD.

Furthermore, how defects in MQC may interact with other sources of neuronal vulnerability in PD is another major gap in knowledge. Other populations of neurons that selectively degenerate in PD, such as the LC, are relatively spared in patients with *PINK1/PRKN* mutations [53, 56], indicating that deficiencies in PINK1/Parkin-mediated MQC are not be the sole determinant of selective vulnerability. Other proposed causes include oxidative stress (eg. loss of iron homeostasis), dopamine toxicity, autonomous pacemaking driving rhythmic Ca^2+^-dependent action potentials, and other vulnerabilities in mitochondrial function arising from the size of axonal arbors (Reviewed in [4] and [13]). Many of these mechanisms are clearly interlinked – such as intracellular Ca^2+^ influx driving mitochondrial Ca^2+^ uptake leading to increased ATP and ROS production [202, 203] – but where MQC deficits sits in the intertwined network of vulnerability factors is unclear. Further exploring the relationships within these networks may reveal key hubs that may prove to be more amenable to disease modifying therapeutics.

Beyond a neuron-centric view of PD pathogenesis, recent and ongoing studies of non-neuronal MQC defects and neuroinflammation will bolster our understanding of non-cell autonomous mechanisms of neurodegeneration. Finally, further elucidating the mechanistic interaction of α-synuclein aggregation and PINK1/Parkin MQC inactivation will be critical for establishing the role of MQC in sPD and synthesizing a more unified understanding of PD pathogenesis.

## Data Availability

Not applicable.

## Abbreviations

(nc-)mtRNA: (nuclear encoded) mitochondrial RNA
CCCP: Carbonyl cyanide *m*-chlorophenyl hydrazone
CNS: Central nervous system
DA: Dopamine
ETC: Electron transport chain
IMM: Inner mitochondrial membrane
LB: Lewy bodies
MDV: Mitochondria-derived vesicle
mitAP: mitochondrial antigen presentation
MPTP: 1-methyl-4-phenyl-1,2,3,6-tetrahydropyridine
MQC: Mitochondrial quality control
mtDNA: mitochondrial DNA
OMM: Outer mitochondrial membrane
OTC: Ornithine transcarbamylase
PARIS: Parkin Interacting Substrate
PD: PARKINSON’S disease
PFF: Preformed fibrils
RER: Rough endoplasmic reticulum
ROS: Reactive oxygen species
SNpc: Substantia nigra pars compacta
sPD: sporadic Parkinson’s disease
TOM: Translocase of the outer mitochondrial membrane
